# Putting Stress in Historical Context: Why It Is Important That Being Stressed Out Was Not a Way to Be a Person 2,000 Years Ago

**DOI:** 10.3389/fpsyg.2021.539799

**Published:** 2021-04-20

**Authors:** Fabian Hutmacher

**Affiliations:** ^1^Department of Psychology, University of Regensburg, Regensburg, Germany; ^2^Human-Computer-Media Institute, University of Würzburg, Würzburg, Germany

**Keywords:** stress, modern life, history of psychology, social constructionism, cross-temporal variation

## Abstract

It was not until the middle of the twentieth century that scientists and Western societies began to label the combination of physiological and psychological responses that people display when things are getting too much and out of balance as “stress.” However, stress is commonly understood as a universal mechanism that exists across times and cultures. In a certain sense, this universality claim is correct: the physiological and endocrinological mechanisms underlying the stress response are not a modern invention of our body. In another sense, the universality claim is potentially problematic: stress has become, but has not always been, a way to be a person. That is, the social practices, in which the physiological and endocrinological stress mechanisms are embedded, are not the same across times and cultures. Crucially, these social practices are not a negligible by-product, but form an essential part of the way stress is commonly understood and experienced. Against this background, one may still decide to use the word “stress” when speaking about other times and cultures. Nevertheless, one should at least be cautious when doing so for three reasons. First, using the word “stress” when referring to societies different from our own may create the impression of a similarity between then and now, which does not actually exist. Second, it may blind us to the nature of the differences between times and cultures. Third, it naturalizes a contemporary scientific concept, which is more adequately viewed as the result of complex social, historical, and societal processes. Putting the stress concept in historical context and acknowledging that its use emerged in a specific historical environment enables us to take a step back and to think about the ways that stress shapes our lives. In other words, viewing stress as a culture-bound concept can give us the possibility to reflect upon our modern societies, in which the concept emerged.

## Introduction

It is hard to determine precisely when the history of the stress concept begins (for an overview, see e.g., Kugelmann, 1992; Doublet, 2000; Cooper and Dewe, 2004; Jackson, 2013). Predecessors like George M. Beard’s “neurasthenia” or Walter B. Cannon’s idea of “homeostasis” can be traced back to the end of the nineteenth and beginning of the twentieth century (Beard, 1869; Cannon, 1929). Note, that the “godfather of stress,” Hans Selye, did not use the term stress in his first publication about the general adaptation syndrome (GAS; Selye, 1936). Although the GAS is generally considered the starting point for the development of his stress theory, it was not before the 1950s that stress was regularly adopted as a scientific term. As Kugelmann (1992, p. 54) put it: “Before the war [i.e., the Second World War], no one spoke of stress; after it, increasingly, everyone did.” However, what is stress believed to be, after all?

This question is not easy to answer as stress is not a narrow term with a fixed meaning: “Stress is a term that causes psychologists headaches. There is a great deal of controversy over what the term means” (Martin et al., 2013, p. 682). Hans Selye, for instance, changed his definition of stress several times (Viner, 1999; Jackson, 2013). More generally speaking, stress is either seen as an aversive *stimulus* we are exposed to, as the *response* we show in reaction to this stimulus or as an *interactional process*, assuming that it is the “lack of fit between the environment and a person” (Mulhall, 1996, p. 459) that makes certain situations stressful. Apart from that, the concept of stress connects research findings from genetics, biology, medicine, and psychology and relates them not only to the individual but also to institutional and societal structures. Thus, stress seems to be a very broad, notoriously fuzzy, and versatile, a “protean concept” (Becker, 2013, p. 2) – a concept that is able to “incorporate a wide range of themes” (Pollock, 1988, p. 387). Put differently, the way that the stress concept is defined and investigated depends on the specific approach and the interests of the individual researcher.

However, this overall impression does not help to answer the question as to whether there is at least some consensus about what stress actually is. What might help is to look at various introductory books to psychology and to see how stress is defined there. Such an approach does not necessarily reveal the “truth” about stress. As textbooks provide a condensed summary of the current academic discourse, they can nevertheless give us a good impression of the way stress is commonly understood in contemporary psychology (see e.g., Smyth, 2004, for an elaboration of how stress as a concept emerged in psychology textbooks). Interestingly, the definitions used in the textbooks seem to share three crucial features.

First, all of them roughly describe stress as the notion that *things are getting too much and out of balance*. People are stressed out because of “specific events or chronic pressures that place demands on a person or threaten the person’s well-being” (Schacter et al., 2014, p. 550; for similar descriptions, see Eysenck, 2009, p. 87; Martin et al., 2013, p. 682; Ciccarelli and White, 2018, p. 462). Stress “is the pattern of response an organism makes to stimulus events that disturb its equilibrium and tax or exceed its ability to cope” (Gerrig, 2013, p. 333). In other words, “[s]tress occurs when aspects of the environment overwhelm people. That is, people feel stressed when too much is expected of them, or when events seem scary or worrisome” (Gazzaniga and Heatherton, 2003, p. 336).

Second, the individual’s response to stress is viewed as a complex pattern, which “is made up of a diverse combination of reactions taking place on several levels, including physiological, behavioral, emotional, and cognitive” (Gerrig, 2013, p. 333; similar descriptions can be found in Gazzaniga and Heatherton, 2003, p. 334; Eysenck, 2009, p. 87; Schacter et al., 2014, p. 550; Ciccarelli and White, 2018, p. 462). As Martin et al. (2013, p. 682) puts it: “In general terms, stress is a pattern of physiological, behavioural, emotional, and cognitive responses to real or imagined stimuli that are perceived as preventing a goal or endangering or otherwise threatening well-being.” Expressed differently, the *inseparable combination of physiological and psychological factors* is what constitutes stress.

Third, stress is believed to be “a common component of everyday emotional life” (Gazzaniga and Heatherton, 2003, p. 334) as “[e]very organism faces challenges from its external environment and from its personal needs” (Gerrig, 2013, p. 332), ranging from major life events, such as a natural catastrophe or the death of a close family member to daily hassles, which are all those “personal events that affect the comfortable pattern of our lives and little annoyances that bug us day after day” (Schacter et al., 2014, p. 550). Expressed more philosophically: “Life is really about change. Every day, each person faces some kind of challenge, big or small” (Ciccarelli and White, 2018, p. 462). From the perspective of evolutionary psychology, one might add the following: “Stress is not a direct product of cultural evolution but is a product of natural selection. It is a behavioural adaptation that helped our ancestors to fight or to flee from wild animals and enemies” (Eysenck, 2009, p. 87; Martin et al., 2013, p. 682). Thus, stress appears as a *universal mechanism*, which is not restricted to certain times and cultures.

If stress indeed is a universal mechanism consisting of a combination of physiological and psychological responses that people show when things are getting too much and out of balance, one could ask how stress levels have developed over the course of time and whether people living in our modern era are more stressed out than they used to be in pre-industrial societies. The first section (“The Stress of Life”) addresses this question by sketching out different lines of reasoning. However, the second section (“Being Stressed Out as a Way to Be a Person”) attempts to explain why comparing the hardships of the past and the present in order to conclude whether ancient or contemporary lives are more stressful is potentially problematic. Building on the framework suggested by Hacking (2007), I argue that being stressed out has become, but has not always been, a way to be a person. If this is the case, the assumption that stress is a *universal mechanism* seems questionable. Stress may be universal in the sense that people from all times and cultures (at least sometimes) have the experience that things are getting too much and out of balance. However, the way that people deal with this experience, that is, the way that people connect physiological and psychological responses, differs across times and cultures, making the stress concept highly context-dependent. This distinction is elaborated in the third section (“Stress as a Universal Mechanism?”). The final section of the paper (“The Importance of Putting Stress into Context”) discusses three reasons why we should be cautious when using the term “stress” in order to analyze the past. In short, the concept of stress can only be properly understood when put in historical context. In addition, such a contextualization may also help us to understand the functioning of our present-day societies.

## The Stress of Life

Assuming that stress is a universal mechanism, that is, that stress exists across times and cultures, asking how stress levels vary across times and cultures is a sound question – and it in fact is a question posed by various authors. The answers that these authors give differ fundamentally and can be sorted into two groups: those that point to processes of acceleration and flexibilization in modern societies and those that point to the hardships and uncontrollable risks that existed in pre-industrial times. While the first group holds that modern life leads to increased stress levels, the second group believes that life was unimaginably harder in pre-modern societies. Let us consider these two types of answers.

For those who believe that being stressed out is deeply intertwined with the way our modern societies work (e.g., Sennett, 1998, 2006; Rosa, 2010, 2013), ours is an age of stress. It is argued that “[m]odern industrialized society sets a rapid, hectic pace for living. People often have too many demands placed on their time, are worried about uncertain futures, and have little time for family and fun” (Gerrig, 2013, p. 332). Hence, stress is regarded as the result “of living in the fast lane” (Jackson, 2013, p. 15), as an inescapable consequence of our lifestyle “with its multiple demands, little time for relaxation, and pressure to achieve” (Donnelly and Long, 2003, p. 398). In line with that, “the body’s responses to stress are [sometimes] regarded as being entirely appropriate, but inadequate to meet the intolerably high and relentless demands imposed by modern living conditions” (Pollock, 1988, p. 388). Expressed in more dramatic words this would mean, that the “[c]ultural progress, having outpaced biological evolution, invalidates some of the wisdom of the body” (Kugelmann, 1992, p. 34). In pre-industrial societies, people did not work in offices in which they were supposed to prepare a presentation for the next meeting while simultaneously helping their colleagues, answering mails, picking up the phone, talking to their boss who has just entered the room, and ignoring the radio which is playing in the background. Back then, people did not have a family with both spouses working full-time, being expected to be deeply in love with each other and being caring parents for their children, managing the household, keeping in touch with their friends, and living in peace with the neighbors who always complain about the kids being too noisy. Back then, people did not live in a world in which they were bombarded with information, making it tremendously difficult if not impossible to distinguish the important things from all the white noise. In short, it is the pace and the demands of our hectic, modern life that “stress us out.”

Although this claim seems to reflect a common belief, it does not remain unquestioned. Pollock (1988) writes, for instance, that “it is hard to see on what grounds we could be justified in assuming that the populations of non-industrial societies carry a lighter burden […]. These communities have their own fair share of morbidity and mortality, though these differ from our own” (p. 388; see also Jackson, 2014). Admittedly, the reply might go on, there were no smartphones, tablets, and computers back then, no newspapers and no television, no cars and no airplanes to make us feel rushed and overwhelmed. On the other hand, these technologies have diversified and enriched our lives to an incredible extent. Following this logic, the complexity of our modern societies is not a sign of decadence, but of security, wealth, happiness, and improved health. In line with that, Eysenck (2009, p. 87) argues as follows:

It is probably true that more people than ever *report* being stressed. However, it isn’t clear that most people are more stressed than used to be the case. Our ancestors had to contend with major epidemics, poor life expectancy, poverty, and an almost complete absence of holidays. Taking all that into account, my hunch is that stress levels nowadays are much the same as in the past.

In fact, one may take an even more radical stance regarding this question: compared to the daily struggle to survive in the face of crop failures, famines, epidemic diseases, and natural disasters, one might say, our modern complaints about multiple demands and the increased pace of life are almost ridiculous. If anything, life was not easier but unimaginably harder in the past. If anything, people back then were far more stressed out than we are today.

So who is correct: those who claim that modern life leads to increased stress levels or those who claim that life was unimaginably harder in pre-modern, pre-industrial societies? As I shall argue in the following, it is almost impossible to answer the question satisfactorily. In contrast to what the cited authors seem to believe and in contrast to what contemporary psychology textbooks claim, it is far from clear that – or at least: *in what sense* – stress is a universal mechanism. Elaborating on this idea is the topic of the next section.

## Being Stressed Out as a way to be a Person

Hacking (2007) has noted that a “scientific classification may bring into being a new kind of person, conceived of and experienced as a way to be a person” (p. 285). He named this process *making up people* and added a second term, the so-called *looping effect*, referring “to the way in which a classification may interact with the people classified” (p. 286). Hacking considers neither the development of a scientific theory nor the process of making up people to be arbitrary. He solely points out that using a system of classification never remains without consequences. When classifying people, we most likely also create *institutions*, which are responsible for classifying and dealing with the classified. Furthermore, we start to produce and to spread *knowledge* about the people we have made up, not to forget that there will be trained *experts* in the field as well. Finally, the possibility *of being categorized* enables people who have been categorized to act and behave differently. This might then, in turn, change the way the system of classification works. As MacIntyre (1985, p. 898) has put it in a similar context, the impact of theories on human thinking and behavior is twofold: “it provides new models for self-knowledge *and* a partially new self for us to have knowledge of.”

In his paper, Hacking is mainly concerned with autism and multiple personality disorder, that is, with quite stable social categories and identities (for applications to other psychological phenomena, see, e.g., Thompson, 2017; Strle and Markič, 2018). In comparison to these phenomena, stress appears to be a rather temporary psychological state. For instance, it is perfectly sound to say that one was not stressed out yesterday but that one is stressed out today. In contrast, one would normally not claim that one is a person with autism today but not tomorrow. Thus, one may ask whether Hacking’s framework can be transferred to analyzing the concept of stress. I believe that the answer is positive. First, one may point to the fact that many people are not only stressed out every now and then but also experience stress regularly if not chronically. For those people at least, the state of being stressed out seems permanent rather than merely transient. Second, even if one were convinced that being stressed out reflects a temporary psychological state and not a stable identity, one would have to keep in mind that the concept of stress is a well-established social category that one can easily refer to if necessary. In other words, while the psychological state of the individual may be variable, the existence of the category itself is not. The concept of stress is deeply rooted in our present-day Western societies and can be used to describe one’s feelings, thoughts, and experiences. Just consider how stress shapes our daily lives:

People use a stressful day at work as an excuse not to tell their children their bedtime story or as an explanation why they are always fighting with their partner. They participate in stress management seminars in order to learn relaxation techniques and coping strategies, ask their doctors to put them on sick leave, and talk to their psychotherapists. Stress is also used to state the discomfort with current societal and economic developments (Hutmacher, 2019, p. 181).

Moreover, we can frequent institutions, which are specialized in treating the stressed, make an appointment with experts in the field who have the necessary knowledge to help us out, and we can use the term “stress” to denote what bothers us. Most importantly, others understand our complaints. Everyone knows what it feels like to be stressed out. Thus, stress “keeps consultants busy, researchers productive, exercise instructors jumping, and ordinary citizens experimenting with an increasingly complex array of diets, life-styles and technological stress-reducing gadgets” (Kugelmann, 1992, p. 21; see also Becker, 2013). For us, being stressed out is a way to be a person.

However, what about other times and cultures? Consider for this purpose the following two statements (adapted from Hacking, 2007, p. 299):

- There were no stressed people in 500 BC; there are many stressed people in 2021.
- To be stressed out was not a way to be a person in 500 BC, people did not experience themselves in this way, they did not interact with their friends, their families, their employers, their counselors, in this way; but in 2021 this is a way to be a person, to experience oneself, to live in society.

In line with Hacking (2007), I argue that B is true whereas A can be either true or false depending on how we read it. A is false in the sense mentioned above: no matter when and where people lived, they always had to carry their burdens – in 1900 as well as in 2021 or 500 BC. However, if A is seen as a brief version of B, it is true. B reflects the idea that a scientific concepts such as stress cannot be understood properly without taking into account the historical and societal circumstances in which its development and use is embedded.

In other words, the stress concept cannot be described adequately without referring to the social practices it is involved in. This means that a “stressed subject is different from one without such a qualifier: she or he can be treated or behave differently” (Bicknell and Liefooghe, 2006, p. 381). In 500 BC, people could *not* use a stressful day at work as an excuse not to tell their children their bedtime story or as an explanation why they are always fighting with their partner. They did *not* participate in stress management seminars in order to learn relaxation techniques and coping strategies. They did *not* ask their doctors to put them on sick leave, and they did *not* talk to their psychotherapists.

## Stress as a Universal Mechanism?

Where does this leave the idea that stress is a *universal mechanism*? Based on the framework provided by Hacking (2007), the answer seems straightforward: being stressed out has become, but has not always been, a way to be a person. Put another way, “stress” as a social practice is a deeply culture-bound product of our modern Western societies and *not a universal mechanism*. However, one could try to defend the notion that stress indeed *is a universal mechanism* by pointing to the fact that the physiological and endocrinological mechanisms constituting the stress response are not a modern invention of our body. This counterargument deserves serious consideration.

Simply put, if we experience a situation in which “things are getting too much and out of balance,” our body shows a wide range of hard-wired responses. For instance, driven by an activation of the sympathetic nervous system, blood pressure, heart rate, and glucose levels increase, while reproductive and digestive functions are reduced (for an introductory overview, see, e.g., Karemaker, 2017). In addition, the hypothalamic-pituitary-adrenocortical axis (HPA-axis), a neuroendocrine system, which has already been investigated by Hans Selye, is activated and releases as cascade of hormones (for an overview, see, e.g., Tsigos and Chrousos, 2002; Charmandari et al., 2005; Smith and Vale, 2006). Moreover, prolonged chronic stress has been associated with various diseases such as depression and cardiovascular disease (e.g., Cohen et al., 2007, 2016) as well as dysregulations of the immune system (e.g., Morey et al., 2015). Against this background, it seems tempting to dismiss the social practices, in which these physiological and endocrinological stress mechanisms are embedded, as a negligible by-product and to conclude that stress *essentially* indeed is a universal mechanism.

From my point of view, this conclusion would be too simplistic. The idea that it means something for us as human beings to be stressed out, that is, that being stressed out is a way of understanding ourselves, is based on the assumption that stress is not only a physiological, but also a psychological construct. Put differently, the way stress is commonly understood is based on the idea that the psychological responses to stress and the social practices involved in coping with stress are more than a negligible by-product. However, one may ask, would it not be possible to change this definition and to reconceptualize stress as a purely physiological and endocrinological mechanism? Such an approach would run into practical and theoretical problems. Successfully enforcing such a redefinition of the stress concept seems *practically* impossible, as the way stress is understood in the public as well as in the scientific discourse, it is far more than a purely physical condition. As described above, conceptualizing stress as the inseparable combination of physiological and psychological factors is deeply rooted in our present-day societies. So even if a researcher decided to apply the term stress to physiological or endocrinological mechanisms only, the researcher would constantly run at risk of being misinterpreted, as the researcher’s definition of stress would conflict with the way the concept is commonly applied.

However, there is an even more fundamental theoretical argument: stress does not exist as a purely biological state in humans – and it never can. This claim can be illustrated using an analogy about “good parenting” proposed by Thompson (2017, p. 55):

Being a good parent consists in a host of emotional and cognitive skills and putting those skills into play in action. The skills and the behaviors based on them clearly depend on the brain – and improving them changes the brain – but they are not private mental states and do not exist inside the brain. Although it is possible that unique patterns of brain activity correlate with being a good parent in a given context, appealing to their presence would not explain what it is to be a good parent. Parenting does not exist inside the brain; it exists in the social world of human life. Furthermore, what counts as good parenting depends on the social context and the culture. So parenting simply is not visible at the level of the brain. To bring it into view, we need a wider perspective, one that takes in the context of the whole person as well as the social and cultural environment.

The same applies to stress: stress clearly depends on certain innate biological mechanisms, but it is not a private mental state and it does not exist inside the brain. Stress is unthinkable without the manner in which we relate to our body’s reactions, the way we frame, interpret, guide, and organize them (see Kugelmann, 1992, p. 2). This also means that the social practices, in which the physiological and endocrinological stress mechanisms are embedded, form an essential part of the way stress is commonly understood. As discussed above, however, the manner in which we do relate to our body’s reactions as well as the manner in which we integrate these reactions into our scientific and societal discourse is not hard-wired: It is the result of complex social, historical, and societal processes. The fact that being stressed out has become a way to be a person simply means that our contemporary (Western) societies have developed a rule-governed, intersubjectively understandable, and institutionalized way of relating humans’ thoughts and behavior to a certain kind of bodily experience.

So is stress a universal mechanism? Yes and no. Yes, because the physiological and endocrinological mechanisms underlying the stress response are the same across times and cultures; and no, because the social practices in which these physiological and endocrinological mechanisms are embedded, have changed over time: being stressed out has become, but as not always been, a way to be a person. As the social practices surrounding the biological basis are not a negligible by-product of stress, but form an important part of the way stress is commonly understood, using the term “stress” when speaking about other times and cultures may be problematic. In other words, using the term “stress” is likely to create associations about institutions and experts, about the creation of knowledge and research programs, and about ways of interacting with others, which cannot be transferred directly to societies different from our own. Before discussing the potential consequences of this conclusion, I want to illustrate the point that I have tried to make by taking a closer look at two archeological studies.

### The Iceman

The mummified body of the Iceman (also called “Ötzi”) who lived about 5,300 years ago was discovered in September 1991 in South Tyrol. Since then, his body has been analyzed in order to learn as much as possible about the circumstances of his life and death (see e.g., Seidler et al., 1992; Handt et al., 1994; Capasso, 1998; Macko et al., 1999). Although the mummy had lost all nails post mortem, as has been observed in other ancient mummies as well, one fingernail was found during the archeological excavation. The study by Capasso (1994) is concerned with analyzing this fingernail. In the abstract, the author writes:

[T]he presence of three sets of Beau’s lines have [*sic*] allowed us to determine the seriousness, time of onset, and duration of three periods of intense stress that the subject underwent during the last months of his life […]. […] [T]hey occurred four, three, and two months before death. The final episode was the most serious, and the systemic upheaval it produced lasted at least two weeks (Capasso, 1994, p. 123).

So what are Beau’s lines? First described by Beau (1846) from whom their name is derived, Beau’s lines are the expression “of temporary suffering on the part of the cells that produce onycokeratin. This suffering results in a more or less marked reduction in the growth of the nail, whose thickness is also reduced during the period of stress” (Capasso, 1994, pp. 127–128). As discussed in the literature, Beau’s lines can be caused by both localized pathologies and systemic causes. As “only fairly serious systemic diseases are capable of producing the degree of suffering of the nail matrix necessary to produce a reduction in the growth rate of the nail that can be observed macroscopically” (Capasso, 1994, p. 128) and as the Beau’s lines on the Iceman’s fingernail are very distinct, systemic causes seem to be most likely. Among others, possible systemic causes for the development of Beau’s lines in adults are zinc deficiencies (Weismann, 1977), parathyroidal disease (Lang, 1981), and serious events during which a person’s life was under serious threat (Beaven and Brooks, 1984). No matter what kind of systemic cause may have led to the formation of the Beau’s lines on the Iceman’s fingernail, they seem to be, as Capasso argues, “the result of generic forms of stress” (p. 129).

Is this conclusion correct? First, it is important to note that I do not want to cast doubt on the scientific worth of the cited study. Gaining new insights in the living conditions of the Iceman using the latest instruments and techniques of analysis sounds promising and has indeed yielded impressive results. Second, using the term “stress” in this context may be potentially misleading. Given the results of the discussed study and the findings of other researchers, it seems safe to assume that the last weeks and months in the life of the Iceman were full of deprivation. Nevertheless, the collected evidence does not tell us anything about the *subjective experiences* the Iceman had during this last period of his life. We do not know anything about his thoughts or feelings. We do not know how he as an individual related to the symptoms he experienced. We do not know how he reacted to the diseases he may have been afflicted with. He may or may have not viewed them as a punishment by gods or spirits. He may have suffered from them, but he may also have accepted them as a burden he had to carry. We simply do not know the answer to these kinds of *psychological* questions and there is no way of finding out. However, what we do know is that in the times of the Iceman, there was no health care system comparable to ours. As mentioned above, there were no psychotherapists, no stress-management seminars teaching coping strategies, and no doctors putting exhausted individuals on sick leave. Most certainly, the Iceman had his own strategies on how to deal with fear and despair, misfortune and disease, and most certainly these strategies were not the strategies we use today. The Iceman did not talk about *stressors* and *coping* in the sense that we do today. Simply put, being stressed out was not a way to be a person for the Iceman. He neither experienced himself nor interacted with his contemporaries in this way.

Taken together, using the term “stress” in the context of the Iceman is potentially misleading as it suggests a knowledge about the subjective experience of the Iceman, which we do not have, and as it creates the impression of a similarity between his time and our time, which does not necessarily exist. Of course, one could defend the use of the word “stress” in the context of the Iceman by claiming that one was referring to the underlying physiological and endocrinological mechanisms, which we share with the Iceman. In a certain sense, this defense is perfectly sound. Nevertheless, the defense is also incomplete as it discards the social practices surrounding the biological basis of stress too quickly. That does not necessarily imply that we should stop using the word “stress” when speaking about other times and cultures, but it does imply that we should use the word with caution. At least, we should be aware of the fact that there are fundamental differences between *then* and *now*, which are not captured by the umbrella term “stress.”

### Archeological Hair From Peru

Similar lessons can be learned from another archeological study, conducted by Webb et al. (2010) who tried to assess “systemic stress” of long-deceased individuals. In order to do so, the authors “selected hair samples from ten individuals from five different archaeological sites in Peru, and analyzed them in segments to determine cortisol levels” (p. 807). The bodies of the investigated individuals were between 500 and 1,500 years old. The authors hypothesized that the “[a]nalysis of cortical levels in archaeological hair should enable assessment of stress during a short, but critical, period of an individual’s life” (p. 807). Indeed, the hair samples of all individuals “showed measurable levels of cortisol” (p. 809). Thus, “individual experiences of stress can be reconstructed for the period of time represented by each hair sample” (p. 807). Furthermore, “[t]he segmental data suggest that stress in the months and years represented by our hair samples is highly variable among individuals, and it is likely that the causes of that stress are also diverse” (p. 811).

Again, my reservation is not about the idea of trying to understand more about the life of deceased individuals about whose lifestyle and living conditions we have no source except from their mortal remains; my reservation is not about the data itself, but about the way they are *presented*. The cortisol found in the hair of the 10 investigated individuals may in fact represent their bodies’ reaction to the demands of their environment. In this context, the observation that their “[c]ortisol levels are somewhat high compared to modern healthy individuals […] [and that] this is not entirely unexpected because these individuals were approaching death and some may have been chronically ill” (p. 810) sounds reasonable, too. My point is simply that we have no idea how people dealt with these struggles and difficulties in the societies or communities they belonged to. Thus, using the term “stress” to denote what the individuals were bothered with can be seen as a simplification. In the lack of better options, we may still decide to do it. However, we should at least be aware what we are doing.

## Discussion: the Importance of Putting Stress Into Context

As the first section of the present paper has illustrated, contemporary psychology commonly views stress as a *universal* mechanism consisting of a combination of physiological and psychological responses that people show when things are getting too much and out of balance. In a certain sense, this universality claim is correct: The physiological and endocrinological mechanisms underlying the stress response are not a modern invention of our body, but have remained the same across time and cultures. In another sense, the universality claim is potentially problematic: neither for the Iceman nor for the 10 individuals, who died in South America between 500 and 1,500 years ago, “being stressed out” was a way to be a person. The use of the stress concept is in fact restricted to certain times and cultures. More specifically, the social practices in which the physiological and endocrinological stress mechanisms are embedded have changed over time. Hence, using the term “stress” when referring to the burdens people from different times and cultures had to carry can be seen as an oversimplification. However, one may still decide to use the term “stress” when referring to other times and cultures for *pragmatic reasons*. While the terms that the communities under investigation have used to describe their burdens may be unknown or at least foreign to us, “stress” is a commonly understood concept. Hence, one could argue that using “stress” as an umbrella term that helps our understanding of the sorrows and worries of the past as well as connects us to cultures and civilizations that have long since disappeared. While I see the merits of such a pragmatic approach, the last section of the present paper is dedicated to exploring why I believe that we should be cautious when using the stress concept to understand the past. In my opinion, there are at least three reasons, some of which I have already briefly mentioned above. First, using the term “stress” when referring to past times and cultures is potentially problematic, because it may create the impression of a similarity between *then* and *now*, which does not actually exist. Second, it may blind us to the nature of the differences between times and cultures. Third, it naturalizes a contemporary scientific concept, which is more adequately viewed as the result of complex social, historical, and societal processes. These three reasons shall be discussed in more detail.

Culture shapes our lives and it shapes them profoundly. It shapes the way we think (e.g., Nisbett, 2003), feel (e.g., Osgood et al., 1975; Russell, 1991), and perceive (e.g., Segall et al., 1966). It shapes our hopes, our fears, and our sorrows. It shapes the things we consider to be desirable and the ones we despise. A brief look at history is enough to realize that different communities have created completely different lifestyles, sets of rules, and moral principles (for a classic but controversial example, see Hofstede, 1980, 1991). For instance, our modern Western concept of identity substantially differs from the concept of identity not only in pre-modern and ancient times, but also in the medieval ages (for a description of the development of modern Western identity see, e.g., Taylor, 1989). As we grew up in our contemporary societies, the way they are designed may feel completely natural to us. Realizing that they are in fact man-made can help us to remain modest. It can free us from the hubris that everyone thinks about the world just as we do. This should not be misunderstood as a plea for relativism: acknowledging the existence of different conceptualizations is rather a starting point for discussions than the end of it (for an overview of the different points of view in the relativism-universalism debate, see, e.g., Berry et al., 2011, pp. 6–8). “Stress” as a scientific concept was developed in Western, that is, industrialized, wealthy, and democratic societies – to name just a few superficial keywords (for the problem of “weird” samples and theories in psychology, see, e.g., Henrich et al., 2010; Apicella et al., 2020). Most societies of the past were not societies of this kind. Hence, they probably had different ways of operationalizing and discursivizing their hardships. We can compare these conceptualizations, learn from them, and criticize them. Thus, putting stress in historical context can prevent us from getting caught up with our own ideas and perceptions. It can teach us modesty and help us to avoid the pitfalls that come with assuming that past societies essentially worked in *similar* ways to our present-day societies. Additionally, carving out the rules and conventions on which other societies were based may be critical for understanding the concepts they used in order to deal with their daily struggles. It is quite likely that we will not be able to understand the solutions that past societies created for coping with certain problems, or that we will find them extremely odd and hard to grasp, unless we manage to identify the key characteristics of the thinking underlying these solutions. That is, until we understand in which ways their thinking was *different* from our own.

In this context, it has recently been argued that psychology should put more effort into understanding cross-temporal variation (Muthukrishna et al., 2020). This would include analyzing historical data, most likely using a wide range of different methods, which capture the full range of the involved mental, behavioral, and social processes (e.g., Mayrhofer and Hutmacher, 2020). Providing a detailed analysis of historical data to see how other times and cultures have dealt with situations in which “things were getting too much and out of balance” is highly desirable, but far beyond the scope of the present paper. Hence, one fictional example shall suffice to illustrate the interplay of the two aspects discussed so far. Imagine a deeply religious peasant in the Middle Ages who has just experienced a crop failure (for a nuanced description of popular religion in the Middle Ages, see, e.g., Brooke and Brooke, 1984; Geary, 2001). He is desperate as he is expecting a hard winter. He fears that he will be unable to feed his family. His parents, his wife, and his six children will starve or may even die. Hence, he goes to church, lights a candle and prays to God, begging him for mercy. Saying that this peasant is stressed out and trying to cope with his misfortune by turning to God would imply both the attempt to make the peasant similar to us and the inability to see that he is actually doing something quite different. The peasant does not pray to God in order to feel better; turning to God is not a coping strategy for him. Expressed differently, the peasant is not taking an instrumental stance toward God. He is deeply convinced of God’s presence in the world and believes that his fate is in God’s hands. His view of the world and of his place in this world is profoundly different from ours. He believes that turning to God is the only adequate way of reacting to the problems he is facing. While for us, there may be “helpful cognitions” and “reappraisal techniques,” which we can use, for the medieval peasant, there is a fixed world order with eternal rules. While for the most of us, eternal life is a vague possibility at the very most, it is an undisputable certainty for the peasant in the Medieval Ages. While for us expressing our potential and following our personal desires and ambitions is an important part of our lives, the peasant most likely believes in a predetermined societal hierarchy God has given him a fixed place in. Although this is only a rough and oversimplifying sketch, it should have become clear by now that these differences could potentially remain unnoticed when the term “stress” is used uncritically.

So far, “stress” has been presented as a concept that enables us, as modern, Western selves, to experience ourselves and to interact with others in certain ways (see, e.g., Hepburn and Brown, 2001, for an example of how stress is used in everyday settings). The social practices that we have developed to relate ourselves to our bodily experiences are the result of complex social, historical, and societal processes (for a perspective on the connectedness of biological and cognitive processes, see Varela, 1984). That is not to say that these practices are arbitrary or inadequate. Rather, it seems plausible to assume that stress has become “a way to be a person, to experience oneself, to live in society” (Hacking, 2007, p. 299) in our times for very good reasons. One may argue, for example, that the rapid technological and economic transformations in the twentieth and twenty-first century have contributed to making stress a popular concept or have at least helped to reinforce its ongoing success (see e.g., Sennett, 1998, 2006; Rosa, 2010, 2013). One may also argue that the concept of stress is deeply intertwined with the way modern identity is understood in our contemporary Western societies (Hutmacher, 2019). If this is the case, it does not matter that the Iceman did not experience himself as being stressed out. We do. Thus, acknowledging that the social practices, in which the physiological and endocrinological stress mechanisms are embedded, have changed over time, may open up a space for reflection. It may not only help us to understand why feeling stressed out has become a way to be a person in our specific historical context; it may also help us to take a step back and to think about the ways “stress” shapes our lives and whether we consider this to be desirable or not.

## Author Contributions

FH developed the research idea and wrote the manuscript.

### Conflict of Interest

The author declares that the research was conducted in the absence of any commercial or financial relationships that could be construed as a potential conflict of interest.
